# Screening for distant metastases in head and neck cancer patients using FDG-PET and chest CT: validation of an algorithm

**DOI:** 10.1007/s00405-015-3773-8

**Published:** 2015-09-09

**Authors:** Asaf Senft, Otto S. Hoekstra, Birgit I. Witte, C. René Leemans, Remco de Bree

**Affiliations:** 1 Department of Otolaryngology-Head and Neck Surgery, VU University Medical Center, Amsterdam, The Netherlands; 2 Department of Radiology & Nuclear Medicine, VU University Medical Center, Amsterdam, The Netherlands; 3 Department of Epidemiology and Biostatistics, VU University Medical Center, Amsterdam, The Netherlands; 4 Department of Head and Neck Surgical Oncology, UMC Utrecht Cancer Center, University Medical Center Utrecht, P.O. Box 85500, 3508 GA Utrecht, The Netherlands

**Keywords:** Head and neck squamous cell carcinoma, Distant metastases, 18F-fluorodeoxyglucose, Positron emission tomography, Computerized tomography, Algorithm, Screening

## Abstract

In patients with head and neck squamous cell carcinoma and high-risk factors, the combination of whole body FDG-PET and contrast-enhanced chest CT has the highest sensitivity and accuracy when screening for distant metastases. The aim of the present study was to retrospectively validate an earlier developed algorithm for interpreting the combination of screening PET and CT. The test cohort consisted of 47 consecutive HNSCC patients with high-risk factors for distant metastases, who had previously undergone FDG-PET and CT and had a minimum 12 months of follow-up. In 12 (26 %) patients, distant metastases were detected during screening or within 12-month follow-up. In patients with locoregional control during follow-up, the sensitivity and specificity were 55 % (95 % CI 23–83 %) and 97 % (95 % CI 82–99 %), respectively, for chest CT, 55 % (95 % CI 23–83 %) and 100 % (95 % CI 88–100 %), respectively, for PET and 73 % (95 % CI 39–94 %) and 100 % (95 % CI 88–100 %), respectively, for the combination of PET and CT. The proposed algorithm was considered to have been validated. In this algorithm, all FDG-PET positive scans for distant metastases (regardless of interpretation of a solid lung lesion on CT) and CT scans with suspicious pulmonary lesions of less than 5-mm diameter (regardless of FDG-PET findings) are considered positive for distant metastases.

## Introduction

Head and neck squamous cell carcinoma (HNSCC) accounts for approximately 5 % of all malignant tumors worldwide. Two thirds of the patients with HNSCC present with advanced stage disease. HNSCCs have a proclivity to metastasize to regional lymph nodes rather than to spread hematogenously. Distant metastases usually occur late in the course of the disease and their presence influences prognosis and choice of treatment. Over the last 2 decade, the success of locoregional treatment has improved significantly, which has resulted in a larger number of patients at risk of developing second primary tumors and distant metastases [1].

Patients with HNSCC and distant metastases are generally not considered curable and often receive palliative treatment alone. Therefore, screening for distant metastases is important to avoid unnecessary or inappropriate treatment.

Screening for distant metastases in all HNSCC patients is not routinely performed because the reported prevalence of clinically identified distant metastases is generally considered too low. The highest prevalence is found in patients with advanced stage disease and extensive lymph node metastases [2]. In previous studies [3], we have identified and validated [4] the following high-risk factors for the development of distant metastases: ≥3 lymph node metastases, bilateral lymph node metastases, lymph node metastases ≥6 cm diameter, low jugular lymph node metastases, tumor recurrence (especially regional) and second primary tumors.

**Fig. 1:**
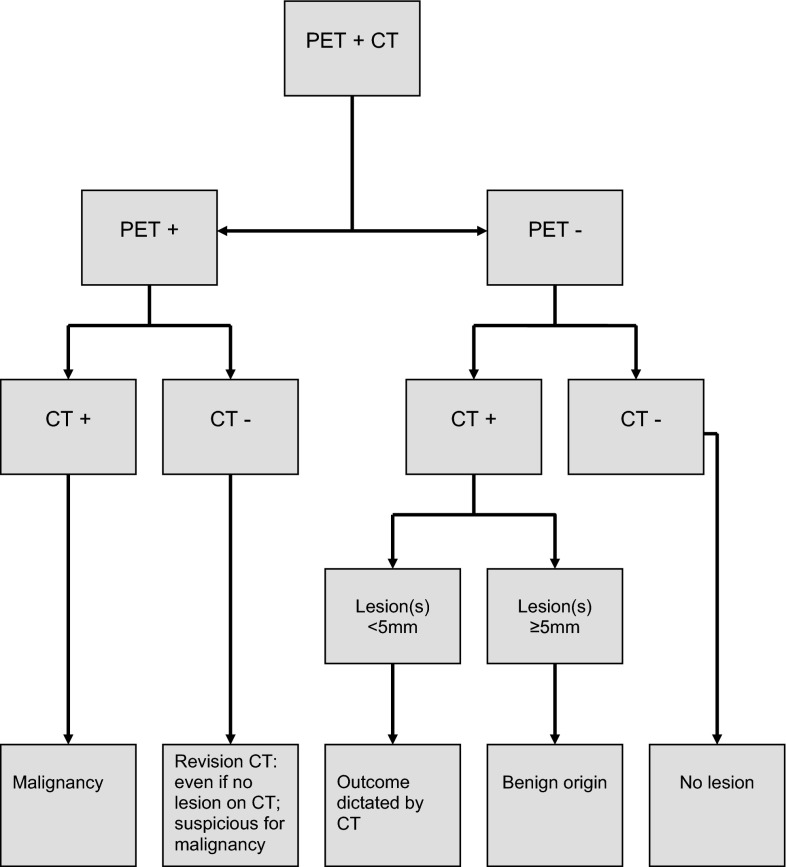
Algorithm for scoring the combination of CT and PET findings for the detection of distant metastases

Positron emission tomography (PET) using the radiolabeled glucose analog ^18^F-fluorodeoxyglucose (FDG) has shown its potential to detect distant metastases [5]. In a prospective multicenter study (SCHOOL), the diagnostic values of contrast-enhanced chest CT (CE-CT) and whole body FDG-PET for the detection of distant metastases were investigated in 92 evaluable patients with the aforementioned high-risk factors [6]. The combination of PET and CT appeared to have the highest sensitivity and accuracy in screening for distant metastases. In addition, the criteria for interpreting the combined PET and CT results were refined using ROC (receiver operated characteristics) curves and logistic regression analysis of the CT and PET results scored using a five-point ordinal scale: if CT and PET are both positive, distant metastasis is very likely to be present; if CT is positive and PET is negative, the final assessment of the combined reading depends on the size of the lesion on CT (for small lesions below the detection limit of PET, outcome is predicted by CT, while for larger lesions PET adds extra information and these lesions are considered negative); if CT is negative and PET is positive, the final assessment of the combined reading depends on the location. The algorithm for lesions based on this previous study is shown in Fig. 1. Because of the current PET detection limit, a 5-mm diameter is used as the cut-off value [6]. To validate this algorithm, we conducted a retrospective cohort study of patients with HNSCC and high-risk factors for dissemination, who had previously undergone screening for distant metastases using whole body FGD-PET and CE-CT of the chest.

## Materials and methods

A single-institution (VU University Medical Center, Amsterdam, The Netherlands) retrospective cohort study of screening for distant metastases tumors with CE-CT of the chest and whole body FDG-PET was performed. Patients who met the following criteria were eligible: (1) HNSCC; (2) candidates for radical treatment with curative intent (surgery and/or radiotherapy with or without chemotherapy); (3) a minimum of 12-month follow-up if no distant metastases or second primary tumor was detected at screening; (4) high-risk factors for the development of distant metastases. Forty-seven patients (35 men and 12 women) with a mean age of 61 years (range 45–86) were identified who met these criteria. They had the following high-risk factors: ≥3 lymph node metastases (*n* = 5), bilateral lymph node metastases (*n* = 23), lymph node metastases ≥6 cm (*n* = 2), low jugular lymph node metastases (*n* = 6), (regional) tumor recurrence (*n* = 5) and second primary tumors (*n* = 16), as assessed by palpation, CT, MRI, and/or ultrasound-guided fine-needle aspiration cytology. Some patients had more than one high-risk factor. Primary tumor sites were the oral cavity (*n* = 11), oropharynx (*n* = 20), hypopharynx (*n* = 7), larynx (*n* = 6), cervical esophagus (*n* = 1) and regional recurrence (*n* = 4). Two patients had synchronous second primary tumors.

### Imaging techniques

All patients underwent CE-CT of the chest and whole body FDG-PET, in an order dictated by logistics. Spiral CT scans were obtained with a fourth-generation Siemens Somaton Plus (Siemens AG, Erlangen, Germany) after intravenous administration of contrast medium (Ultravist, Schering AG, Berlin, Germany). Contiguous axial scanning planes were used with a 5-mm slice thickness without an inter-slice gap. Radiological criteria for: (1) lung metastases were: smoothly defined, sub-pleural suspicious lesions, multiple lesions and lesions located at the end of a blood vessel, and (2) bronchogenic carcinoma were: solitary, spiculated, and centrally located lesions.

FDG-PET was performed after a 6-hour fasting period with ample access to water. At 60–90 min after the intravenous administration of 250–370 MBq FDG, imaging with a trajectory from knee-skull base was performed using a dedicated full ring BGO PET scanner (CTI/Siemens ECAT HR +). Any focal abnormality, which could not be attributed to normal physiological uptake was considered suspicious for malignancy.

### Data analysis

All FDG-PET scans and CT scans were retrospectively scored by one nuclear medicine physician and one radiologist, respectively, with each blinded to the other modality and clinical outcome. For clinical decision making, these scan readings were scored as being either positive or negative for distant metastases. Combined reading of the CT and PET with side-by-side visual correlation was performed by a nuclear medicine physician and a radiologist using the proposed algorithm (Fig. 1).

In all patients (with or without a synchronous second primary tumor) every lesion that was identified was also given a score to indicate how suspicious it was considered to be for a distant metastases. A five-point ordinal Likert scale was used: 1 = definitely benign, 2 = probably benign, 3 = equivocal, 4 = probably malignant, 5 = definitely malignant. If multiple lesions were scored in a single patient, the lesion with the highest score was used for statistical analysis.

The outcome of the clinical diagnostic work-up and the clinical course between screening and when a follow-up period of 12 months had elapsed was used as the reference standard, and patients were classified as positive or negative with respect to the presence of distant metastases. Follow-up was performed by regular visits to the outpatient clinic (every 6 weeks in the first year). During follow-up, the dates of the detection of distant metastases, second primary tumors and/or death were recorded. Although the primary goal was screening for distant metastases, second primary tumors were also registered. Initial screening was classified as true positive if there were evident metastases on chest CT, if lesions on chest CT were progressive or if biopsy (obtained by, for example, bronchoscopy, thoracoscopy, or thoracotomy) revealed metastasis. FDG-PET was considered true positive if a site of increased uptake was proven to be malignant by histopathology obtained using one of the previously mentioned diagnostic techniques. If chest CT or FDG-PET had been abnormal during initial screening, but further pre-operative work-up remained inconclusive, patients were treated as though they had no metastatic disease. If follow-up of 12 months did not reveal metastases, such suspicious CT or FDG-PET results were classified as false positive. If a patient had a negative chest CT or FDG-PET, but developed distant metastases during the 12-month follow-up period, screening was considered to have been falsely negative. Screening by chest CT or FDG-PET was considered true negative if a patient had negative test results and no distant metastases were observed within 12 months.

Patients with negative screening results who manifested distant metastases within 12 months of follow-up were stratified for the presence or absence of locoregional control, because no distinction could be made between growth of subclinical metastases already present at the time of screening and reseeding from a locoregional recurrence. Although the primary aim of screening is to find distant metastases, detection of second primary tumors is an additional, clinically relevant finding. Patients with second primary tumors found during screening or follow-up were analyzed separately.

### Statistical analysis

Sensitivity, specificity and accuracy of CT, PET and the combination of both were calculated with the corresponding exact 95 % confidence interval (CI). Receiver operated characteristic (ROC) analysis was used as an objective measure to evaluate the overall accuracy of CT and PET. The highest Likert score of a suspicious lesion on either CT or PET was used and the level of significance as well as the Q-point (highest sensitivity/specificity) was calculated.

## Results

Pretreatment screening identified distant metastases in 8/47 patients (17 %) and second primary tumors in 3/47 (6 %). All patients with distant metastases were treated with palliative intent. One of the three patients with a second primary tumor had disseminated lung cancer (lung and bone metastases) and was also treated palliatively. The other two appeared to have a second primary with limited stage disease and were treated with curative intent for both the HNSCC and the second primary tumor. In 17 of the total group of 47 patients (36 %) distant metastasis (*n* = 12; 26 %) or a second primary tumor (*n* = 5; 11 %) was detected either during screening or within 12-month follow-up after screening. Both patients who developed a second primary tumor during follow-up also had lung metastases. Since it was impossible to determine on imaging if these metastases originated from the index HNSCC (and were therefore missed by screening) or from the second primary tumor, these patients were not included in the accuracy analysis for the detection of distant metastases. Hence, the accuracy data for the detection of distant metastases were calculated using 45 patients.

### Chest CT

The clinical report of the screening chest CT was positive in 10/47 (21 %) patients. Nine patients had distant metastases (*n* = 6) or a synchronous second primary tumor (*n* = 3). One patient had false-positive findings. Eight of the 37 (22 %) patients with a negative CT-scan at screening developed distant metastases (*n* = 6; 16 %) or a second primary tumor (*n* = 2; 5 %) within the 12-month follow-up period. For the detection of distant metastases CT had (in *n* = 45 patients—see comment above) a sensitivity of 50 % and a specificity of 97 % (Table 1).

**Table 1:** Accuracy of CT, PET and the combination of PET and CT for the detection of distant metastases

Method	Sensitivity	Specificity	PPV	NPV	Accuracy
Percentage with 95 % confidence interval (*n* = 45 patients)					
CT	50 (21–79)	97 (84–99)	86 (42–99)	84 (69–94)	84 (71–94)
PET	50 (21–79)	100 (89–100)	100 (54–100)	85 (69–94)	87 (73–95)
PET and CT	67 (35–90)	100 (89–100)	100 (63–100)	89 (75–97)	91 (79–98)

*PPV* positive predictive value, *NPV* negative predictive value

### FDG-PET

The clinical report of the screening FDG-PET was positive in 9/47 (19 %) patients. All of them had confirmed distant metastases (*n* = 6; 13 %) or a synchronous second primary tumor (*n* = 3; 6 %). Eight of the 38 (21 %) patients with a negative PET at screening developed distant metastases (*n* = 6; 16 %) or a second primary tumor (*n* = 2; 5 %) within the 12-month follow-up period, yielding a sensitivity for the detection of distant metastases of 50 % and a specificity of 100 % (*n* = 45, Table 1).

### CT and PET combined

In the total group of 47 patients, 12 (26 %) patients had either a positive CT or positive FDG-PET. Malignancy was found in 11 (23 %) of these patients; 8 (17 %) distant metastases and 3 (6 %) second primary tumors. CT and PET combined were scored using the aforementioned algorithm. As noted, lesions <5 mm cannot be reliably identified using PET as single screening modality. In these cases, the assessment was predominantly dictated by the CT characteristics.

In the total group of 47 patients, 9 (19 %) patients had a positive FDG-PET. Of these 9 patients, 7 (15 %) also had a positive CT confirming distant metastases (*n* = 4; 9 %) or a synchronous second primary tumor (*n* = 3; 6 %). In the remaining two patients with negative CT, the scans were reviewed. One patient was still considered not to have any lesions, but went on to develop rib metastases during follow-up at the same site where the screening FDG-PET was positive. Another patient had a positive pulmonary lesion with FDG-PET, but CT was scored as negative for metastases. Review of the CT confirmed a lesion of 6 mm, which was scored as being benign. During follow-up, however, distant metastases were subsequently confirmed at this site.

In the total group of 47 patients, 38 (81 %) had a negative FDG-PET. Three of those patients (6 %) had a positive CT and 35 (74 %) patients a negative CT. Of the three patients with a positive CT and negative PET, one patient had a lung lesion of 15 mm, which did not appear to be malignant during follow-up and two patients had multiple lesions of 4 mm which were confirmed during follow-up.

Six of the 36 (13 %) patients with negative FDG-PET and CT developed distant metastases (*n* = 4; 11 %) or a second primary tumor (*n* = 2; 6 %) within the 12-month follow-up period.

For the detection of distant metastases using the combination of PET and CT (*n* = 45), the sensitivity was 67 % and the specificity was 100 % (Table 1).

### Second primary tumors

In 3 of the 47 (6 %) patients, a second primary tumor was found during initial screening while 2 of the 47 (4 %) patients developed a second primary tumor during follow-up. In 3 of the 5 patients, both FDG-PET and CT were true positive for a bronchogenic carcinoma. In the other 2 patients, both FDG-PET and CT were negative during screening.

### Scenario analysis

When only the 40 patients with locoregional control during follow-up were analyzed, the sensitivity to detect distant metastases increased from 50 to 55 % with FDG-PET, from 30 to 55 % with CT and from 67 to 73 % with FDG-PET and CT combined using side-by-side visual correlation (Table 2).

**Table 2:** Accuracy of CT, PET and the combination of PET and CT for the detection of distant metastases in patients with locoregional control

Method	Sensitivity	Specificity	PPV	NPV	Accuracy
Percentage with 95 % confidence interval (*n* = 40 patients)					
CT	55 (23–83)	97 (82–99)	86 (42–99)	85 (68–95)	85 (70–94)
PET	55 (23–83)	100 (88–100)	100 (54–100)	85 (69–95)	88 (73–96)
PET and CT	73 (39–94)	100 (88–100)	100 (63–100)	91 (75–98)	93 (80–98)

*PPV* positive predictive value, *NPV* negative predictive value

### Refined interpretation criteria

**Fig. 2:**
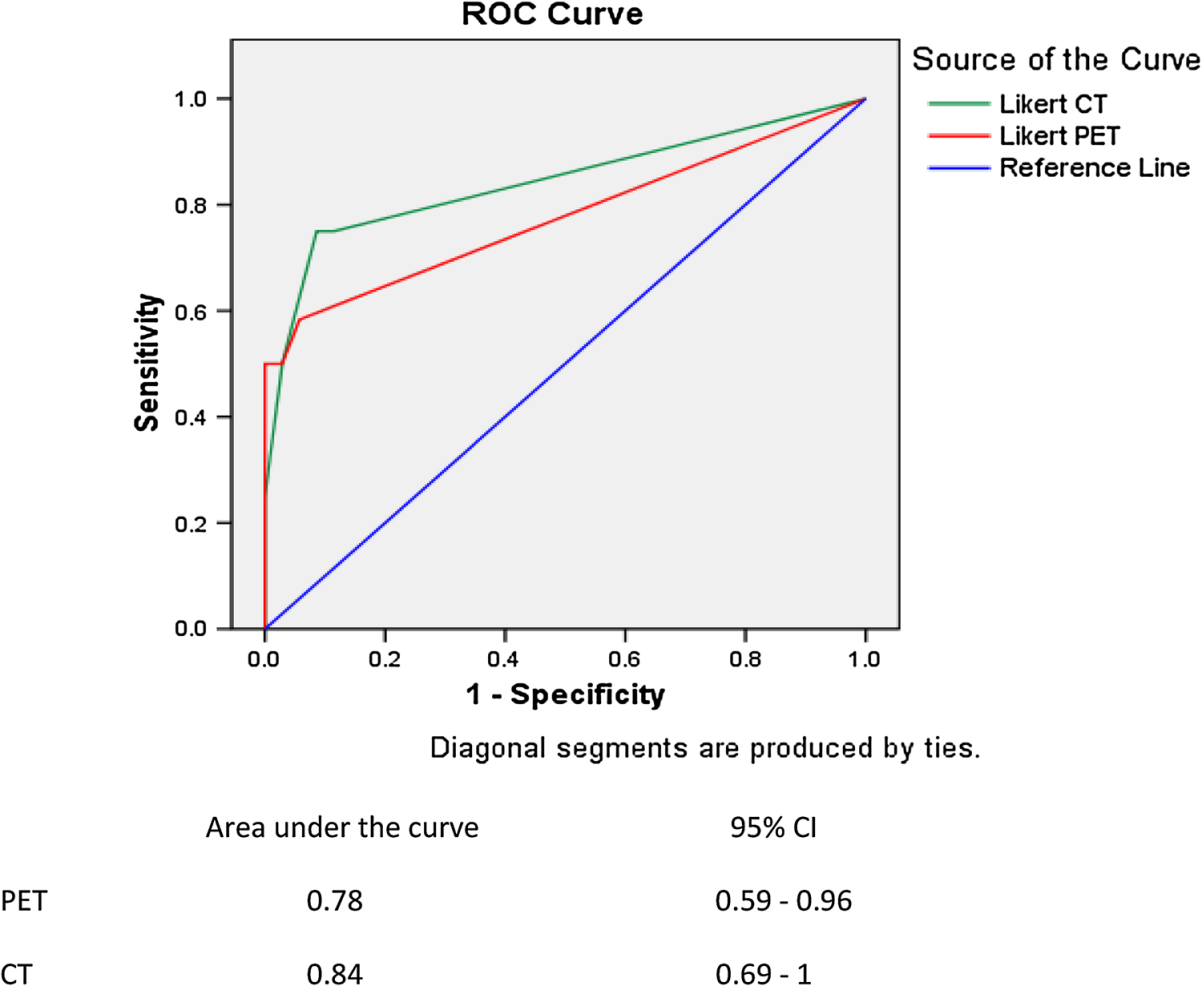
ROC analysis using five-point ordinal classification system for reporting CT and PET results

After all scans were scored according to the five-point ordinal scale for the presence or absence of distant metastases ROC curves were constructed (Fig. 2). If in one patient multiple lesions were scored, the lesion with the highest score was used for statistical analysis. Three patients in which a second primary tumor, but no distant metastases were detected, scored negative (Likert = 0) with respect to the screening for distant metastases. ROC analyses provided areas under the curve (AUCs) of 0.84 and 0.78 for CT and PET, respectively [both significantly different from the null hypothesis (true AUC = 0.5)]. The comparison of both AUCs showed no significant difference (*p* = 0.45). The Q-point for PET was found at a five-point ordinal scale score = 1 for a sensitivity of 58 % and a specificity of 94 %. For CT this point lies at a score = 3 with a sensitivity of 75 % and specificity of 91 %.

## Discussion

FDG-PET and chest CE-CT have good diagnostic performance in detecting distant metastases in patients with HNSCC [7]. However, scoring criteria and interpretation are not well defined, resulting in different study outcomes. In the present study, we validated an algorithm which was based on findings from our previous multicenter study on screening for distant metastases in HNSCC [6].

Using this algorithm on a test set of 47 patients with high-risk factors for dissemination, similar accuracy data for the detection of distant metastases by the combination of FDG-PET and CT were obtained as in the original study. In the group of HNSCC patients with locoregional control a sensitivity of 73 % (95 % CI 39–94 %), a specificity of 100 % (95 % CI 88–100 %), a positive predictive value of 100 % and a negative predictive value of 91 % were found. In the previous study using the same algorithm, these figures were 82 % (95 % CI 65–92 %), 95 % (95 % CI 88–98 %), 86 % (95 % CI 69–94 %) and 93 % (95 % CI 85–95 %), respectively [6].

Regarding clinical relevance, the results of the Q-point and AUCs could suggest that Likert scoring does not add further information to the FDG-PET and that when a lesion is seen on PET, it can mostly be regarded as being malignant. For CT the highest sensitivity is reached when the Likert score is 3 or higher. In our previous studies, the cut-off point was found at Likert 4 or higher [6]. Likert 3 lesions are typically small nodules, which are often the subject of debate regarding benign or malignant origin. The use of Likert scoring can probably not adequately resolve this matter. A substantial interobserver variability in CT interpretation was previously reported [8].

The pre-test probability of the patients and the prevalence of malignant disease influence the optimal scoring criteria and algorithm. The prevalence of malignancy in solitary pulmonary nodules (SPNs) ranges from 5 to 70 % [9]. Since the presence of distant metastases at pretreatment evaluation influences the prognosis and thus treatment selection, detection of distant metastases will alter the treatment plan and may avoid unnecessary or inappropriate treatments which present a burden and risks to the patient, affect quality of life, consume resources and result in costs (e.g., hospital stay, operating time and radiotherapy facilities) [10]. False-positive findings on imaging should have limited clinical consequence since confirmation by histopathology or further imaging is warranted before treatment with curative intent is withheld from a patient. Therefore, in screening for distant metastases sensitivity is to a certain extent more important than specificity.

The extent to which results found by different studies can be generalized, and support the application of CT and PET to this patient group in daily clinical practice, tends to depend on the degree of uniform interpretation using well-defined scoring criteria. CT is extremely sensitive for the detection of pulmonary nodules, but is frequently indeterminate in diagnosis. Increasing numbers of pulmonary nodules are being detected, in large part due to the developments in CT imaging techniques. While specific patterns of calcification or the presence of fat in pulmonary nodules on CT can be used to determine if a nodule is benign, most nodules lack benign characteristics and are, therefore, considered indeterminate for malignancy. Indeed, in a previous study a substantial amount of agreement was found for scoring the presence or absence of malignancy using CT, whereas the agreement was almost perfect using PET [8]. This emphasizes the difficulty in interpreting pulmonary nodules on CT. On PET images lesions are essentially ‘present’ or ‘absent’ which probably makes them less susceptible to variation in interpretation. We have suggested that for optimal assessment in clinical practice one observer is usually sufficient for scoring PET, but CT should probably more often be scored by more than one observer in consensus or combined with PET [8].

If multiple suspicious lesions are detected, malignancy is very likely. Solitary lesions are more difficult to assess. Orlacchio et al. [11] defined indeterminate solitary pulmonary nodules (SPN) as single solid round or oval shape lesions smaller than 3 cm with no unequivocal signs of benign or malignant disease, normally ventilated peripheral parenchyma, absence of hilar or mediastinal nodal enlargement and no extrathoracic findings suggestive of distant metastasis. The assessment of SPN has been studied in different settings: incidental discovery and during the evaluation of cancer patients. Definite criteria for the differentiation of indeterminate SPNs by CT and FDG-PET have not been standardized and are still a matter of debate. Criteria to score an SPN as malignant on CT include location, size, volume doubling time and contrast-enhanced increase in attenuation [11–15].

Scoring criteria for FDG-PET interpretation of an SPN as malignant include hypermetabolic activity greater than the mediastinal blood pool and a (semi)quantitative standardized uptake value (SUV) higher than a certain threshold value [16, 17]. Since different methods to assess the FDG-avidity are used, studies may be difficult to compare [18]. Several studies have found no significant difference between the diagnostic performance of visual interpretation and (semi)quantitative analysis of FDG uptake [16, 17]. Pulmonary lesions with visually absent FDG uptake indicate that the probability of malignancy is very low, while this probability in any visually evident lesion is about 60 % [19]. This supports our recommendation to consider each positive FDG-PET as malignant regardless of the CT interpretation of solid lesions.

Limitations in PET camera resolution hamper the evaluation of nodules less than 8 mm in diameter [19]. In lesions less than 10 mm CT has added value to PET. De Wever et al. [20] found a sensitivity of 100 % for the combination of PET and CT compared to 83 % for PET only in nodules less than 10 mm (the majority were 5–10 mm) in diameter [20]. Fortes et al. [21] found in patients who underwent lung resection for pulmonary metastases from extrathoracic malignancies a significant correlation between the size of the nodule and the sensitivity of FDG-PET: 30 % of the metastatic nodules of 10 mm or smaller were FDG-PET positive, while in nodules larger than 10 mm this figure was about 88 % [21]. A meta-analysis of 1474 pulmonary nodules evaluated by FDG-PET revealed an overall high specificity, but varying sensitivity for nodules less than 1 cm [22]. Other studies also found a higher rate of erroneous FDG-PET results for lesions <10 mm compared to larger lesions [23–25]. However, in indeterminate SPNs greater than or equal to 7 mm PET is more useful than CE-CT due to its high sensitivity and much better specificity [14]. Divisi et al. [26] compared the results of CT and PET/CT in patients with asymptomatic SPN with a diameter between 0.5 and 0.99 cm and between 1.0 and 1.5 cm and found that PET/CT can improve the identification and characterization of potentially malignant pulmonary nodules with a diameter less than 1 cm. In our algorithm, there is an important role for PET for lesions >5 mm.

FDG-PET lacks precise anatomical resolution and may lead to overdiagnosis of some inflammatory conditions. By virtue of its high spatial resolution, CT may serve as a cross-sectional imaging tool complementary to FDG-PET in the evaluation of distant metastases in HNSCC patients and may help to characterize FDG abnormalities. In recent years, dual modality PET-CT has been used to fuse functional PET and morphological CT data in a single examination. Fused 18FDG-PET/CT is increasingly being applied in detecting distant metastases in patients with HNSCC because of its unique capability to image metabolically active lesions and provide more anatomical details than PET only images. Moreover, fusion of FDG-PET and CT may more accurately localize the lesions. The combination of PET and CT by PET-CT is an attractive option, potentially combining the best of both imaging abilities, and providing one combined diagnostic study for the patient.

In conclusion, when screening for distant metastases in HNSCC patients with risk factors for dissemination using whole body FGD-PET and CE-CT of the chest, good performance can be obtained using the proposed algorithm in which all FDG-PET positive scans for distant metastases (regardless of the interpretation of a solid lung lesion on CT) and CT scans with suspicious pulmonary lesions of <5 mm (regardless of FDG-PET findings) are considered positive for distant metastases.
